# Hypermethylation of *BEND5* contributes to cell proliferation and is a prognostic marker of colorectal cancer

**DOI:** 10.18632/oncotarget.22266

**Published:** 2017-11-01

**Authors:** Ruo-Kai Lin, Wan-Yu Hung, Yu-Fang Huang, Yu-Jia Chang, Chien-Hsing Lin, Wei-Yu Chen, Shih-Feng Chiu, Shih-Ching Chang, Shih-Feng Tsai

**Affiliations:** ^1^ Graduate Institute of Pharmacognosy, Taipei Medical University, Taipei, Taiwan, R.O.C.; ^2^ Master Program for Clinical Pharmacogenomics and Pharmacoproteomics, Taipei Medical University, Taipei, Taiwan, R.O.C.; ^3^ Graduate Institute of Clinical Medicine, College of Medicine, Taipei Medical University, Taipei, Taiwan, R.O.C.; ^4^ Institute of Molecular and Genomic Medicine, National Health Research Institutes, Zhunan, Miaoli, Taiwan; ^5^ Department of Pathology, School of Medicine, College of Medicine, Taipei Medical University, Taipei, Taiwan, R.O.C.; ^6^ Department of Pathology, Wan Fang Hospital, Taipei Medical University, Taipei, Taiwan, R.O.C.; ^7^ Professional Master Program in Pharmaceutics and Biotechnology, Taipei Medical University, Taipei, Taiwan, R.O.C.; ^8^ Division of Colon and Rectal Surgery, Department of Surgery, Taipei Veterans General Hospital, Taipei, Taiwan, R.O.C.; ^9^ PH.D Program for Clinical Drug Development of Chinese Herbal Medicine, Ph.D Program in Biotechnology Research and Development, College of Pharmacy, Taipei Medical University, Taipei, Taiwan, R.O.C.

**Keywords:** BEND5, colorectal cancer, DNA methylation, prognostic marker, tumor suppressor genes

## Abstract

Aberrant hypermethylation of CpG islands in tumor suppressor genes (TSGs) contributes to colorectal tumorigenesis. To identify new colorectal cancer (CRC) screening marker, we investigated DNA methylation alterations in novel TSGs. Using HumanMethylation450 BeadChip arrays, CpG regions in *BEND5* were the most highly methylated among all genomic regions in 26 colorectal tumors compared to paired non-neoplastic tissues from a Taiwan cohort. Therefore, *BEND5* was selected for further analysis. Quantitative methylation-specific real-time PCR revealed that 86.7% (117/135) of CRC patients exhibited hypermethylated *BEND5*. Real-time reverse transcription PCR identified that BEND5 mRNA expression was downregulated in 68% (32/47) of the analyzed samples. *BEND5* hypermethylation was associated with poor overall survival (OS) in Taiwan patients with early-stage CRC (*P =* 0.037). In a CRC tissue set from South Korea, OS was higher in patients with high BEND5 protein expression than in those with low BEND5 protein expression (*P =* 0.037) by using immunohistochemistry assays. Consistently, BEND5 hypermethylation was associated with poor OS in patients with early-stage CRC in The Cancer Genome Atlas (TCGA) data set (*P =* 0.003). Multivariate Cox proportional hazards regression analysis further supported that hypermethylation of BEND5 genes was significantly associated with OS in Taiwan and TCGA CRC patients (*P =* 0.023 and 0.033, respectively). Finally, the cell model assay with transient transfection of BEND5 or si-BEND5 knockdown indicated that BEND5 inhibited cancer cell proliferation. In conclusion, epigenetic alteration in the candidate TSG *BEND5* contributes to colorectal cancer development and is a prognostic marker of CRC.

## INTRODUCTION

Colorectal cancer (CRC) is the third most commonly diagnosed cancer worldwide [1] and the third leading cause of cancer death in the United States [2, 3]. In Taiwan, CRC is a major malignancy and the third most common cause of cancer-related death [4]. CRC results from the accumulation of multiple genetic and epigenetic alterations in tumor suppressor genes (TSGs) and oncogenes, which transform normal colonic epithelium into adenocarcinomas [5]. Gene silencing by aberrant DNA methylation of the promoter regions is one of the major roles of epigenetic alterations [6]. Hypermethylation of CpG islands associated with TSGs can cause transcriptional silencing, contributing to tumorigenesis [7–9]. Generally, DNA methylation is not restricted to a single CpG island, but affects multiple independent loci, indicating widespread deregulation of DNA methylation patterns in different tumor types [10–12]. In genomic screening of 98 primary human tumors, an average of approximately 600 aberrantly methylated CpG islands were identified in each tumor [13]. Thus, identifying critical TSGs and DNA methylation markers can facilitate the characterization of various CRC subtypes [7]. In addition, new tools for diagnosis and prognosis and new therapeutic interventions are required in order to improve the clinical outcome of CRC.

The Illumina Infinium HumanMethylation450 BeadChip array has been used to detect genome-wide DNA methylation profile alterations in cancer [14–16]. Using this array, we analyzed 26 paired CRC and noncancerous colorectal tissues and found multiple hypermethylated loci in the promoter and exon 1 regions of *BEND5*. The coding sequence of BEND5 is located on chromosome 1p33 and comprises 1266 nucleotides that encode a predicted protein of 421 amino acids [17]. Aligning BEND5 sequences from UniProtKB (Q7L4P6) revealed one BEN domain within the BEND5 protein sequence. The BEN domain identified in 2008, named after the experimentally characterized proteins BANP, E5R, and NAC1 (BEN), is found in one or more copies in these proteins [18]. Contextual analysis suggests that the BEN domain mediates protein–DNA and protein–protein interactions during chromatin organization and transcription [18, 19]. The BEND5 protein exhibits DNA-binding and repression activities through binding to CCAATTGG or TCYAATHRGAA sequence [20]. Because the precise role of the DNA methylation of *BEND5* in CRC is unclear, we investigated whether the alteration of the BEND5 function is involved in colorectal tumorigenesis.

## RESULTS

### Hypermethylation of the BEND5 promoter and low expression of mRNA and protein in Asian CRC patients

**Figure 1:**
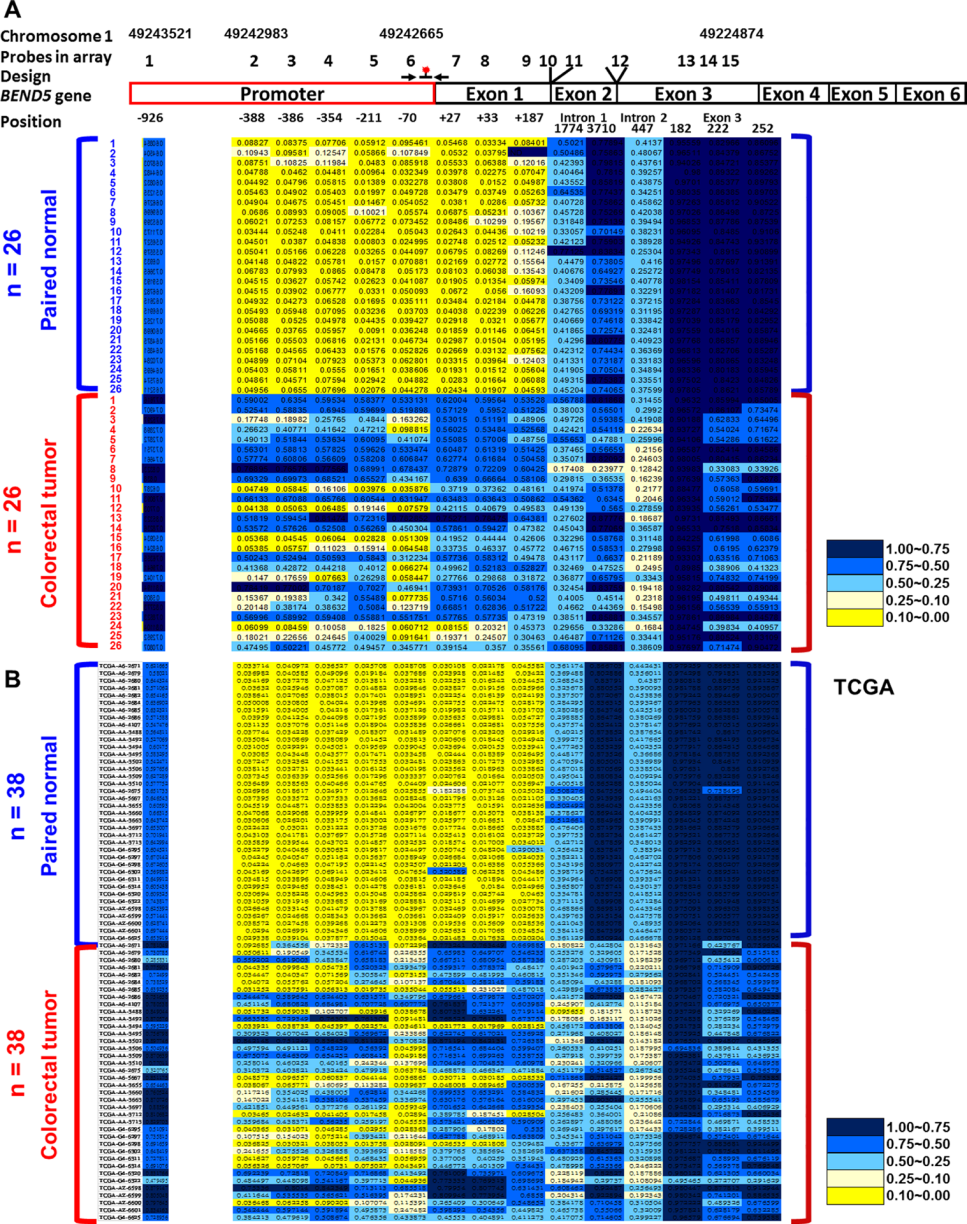
Differentially methylated CpG in *BEND5* in CRC patients Methylation levels (average β values) at the differentially methylated loci were identified using an Illumina Methylation 450K array-based assay in (**A**) 26 CRC patients in Taiwan and (**B**) 38 CRC patients from the TCGA data set. The scale shows the relative methylation status from 0.00 to 1.00 (yellow: hypomethylation, blue: hypermethylation). Fifteen CpG sites on *BEND5* were detected in 26 paired normal (upper) and CRC (lower) tissues. Six CpG sites in promoter regions −926, −388, −386, −354, −211, and −70 are designated 1, 2, 3, 4, 5, and 6, respectively. The CpG sites in exon 1 regions +27, +33, and +187 are designated 7, 8, and 9, respectively. The 1774 and 3710 sites in intron 1 regions are designated 10 and 11, respectively. One site is in the intron 2 region (447) and designated 12. Three CpG sites in exon 3 regions 182, 222, and 252 are designated 13, 14, and 15, respectively. The *BEND5* gene is located on chromosome 1. Positions 1, 2, 6, and 15 are on 49243521, 49242983, 49242665, and 49224874 of chromosomes 1, respectively. Primers and the probe for the QMSP assay are marked and indicated in the junction between the promoter and exon 1 regions.

To identify critical TSGs, the Illumina Infinium HumanMethylation450 BeadChip array was applied to analyze 26 CRC tissues and paired noncancerous colon tissues (Supplementary Table 1). Compared with the matched normal colorectal tissues, the CRC tumor tissues displayed 15 highly methylated sites in *BEND5*, which is the most among all genes. The promoter and exon 1 regions of the BEND5 sequences were significantly hypermethylated (Figure 1A). Therefore, *BEND5* was selected for further analysis, as follows.

The methylation patterns of *BEND5* were verified by performing QMSP assays in 135 CRC patients. Primers and probes were designed for the junction between the promoter and exon 1 region (Figure 1). The data indicated that in 86.7% (117/135) of the CRC patients, hypermethylation of *BEND5* was at least 2-fold higher in the tumor tissues than in the matched normal colorectal tissues (Table 1, Supplementary Figure 1). The status of *BEND5* methylation and expression was similar among the CRC tumors with different clinicopathological features (Table 1).

**Table 1:** Promoter hypermethylation of BEND5 gene in relation to clinical parameters for CRC

Characteristics		Total^a^	Hypermethylation^b^	Low methylation	
		*n*	*n* (%)	*n* (%)	*P*-value^C^
**Total**		135	117 (86.7)	18 (13.3)	
**Clinicopathological parameters**					

**Age**	< 65	58	51 (87.9)	7 (12.1)	1.000
	> 65	71	62 (87.3)	9 (12.7)	

**Gender**	Male	73	65 (89.0)	8 (11.0)	0.450
	Female	62	52 (83.9)	10 (16.1)	
**Tumor type**					
Adeno		127	111 (87.4)	16 (12.6)	1.000
Mucinous		5	5 (100.0)	0 (0.0)	
**Tumor stage**					
I		8	6 (75.0)	2 (25.0)	0.220
II		53	50 (94.3)	3 (5.7)	
III		40	33 (82.5)	7 (17.5)	
IV		30	26 (86.7)	4 (13.3)	
**Primary tumor**					
Submucosa		6	5 (83.3)	1 (16.7)	0.962
Muscularis propria		14	12 (85.7)	2 (14.3)	
Subserosa		97	86 (88.7)	11 (11.3)	
Penetrate the visceral		14	12 (85.7)	2 (14.3)	
**Regional lymph nodes**					
No regional lymph node metastasis		66	61 (92.4)	5 (7.6)	0.117
Metastasis in regional lymph nodes		65	54 (83.1)	11 (16.9)	
**Distant metastasis**					
No distant metastasis		95	83 (87.4)	12 (12.6)	1.000
Distant metastasis		30	26 (86.7)	4 (13.3)	
**Differentiation grade**					
Well		11	10 (90.9)	1 (9.1)	0.507
Moderate		112	99 (88.4)	13 (11.6)	
Poor		8	6 (75.0)	2 (25.0)	
**Vascular invasion**					
No invasion		4	4 (100.0)	0	1.000
invasion		100	89 (89.0)	11 (11.0)	
**Location**					
colon		94	83 (88.3)	11 (11.7)	0.522
Rectal		35	31 (88.6)	4 (11.4)	
both		3	2 (66.7)	1 (33.3)	
**MSI status**					
MSS		50	43 (86.0)	7 (14.0)	0.439
MSI-L		5	4 (80.0)	1 (20.0)	
MSI-H		9	9 (100.0)	0 (0.0)	

^a^For some categories, the number of samples (n) was lower than the overall number analyzed because clinical data were unavailable for these samples.

^b^The BEND5 gene was considered hypermethylated when the methylation level of the BEND5 gene relative to that of the β-actin gene was at least twofold higher in the colorectal tumor compared with the paired normal colorectal tissue sample.

^c^These results were analyzed using the Fisher’s exact test.

**Figure 2:**
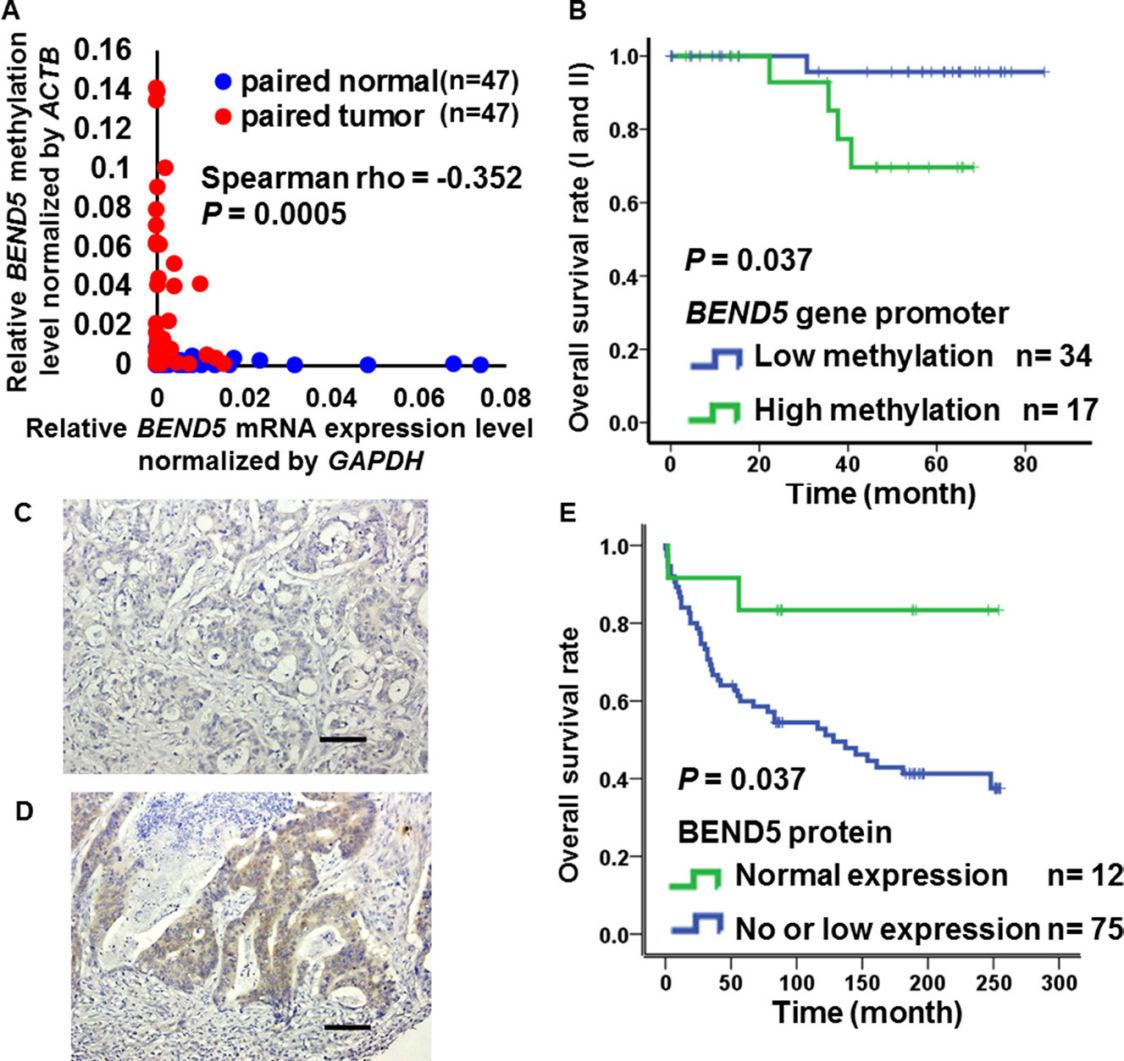
Expression of BEND5 in colorectal cancer (**A**) Kaplan–Meier survival curves were constructed to compare overall survival between CRC patients with low and high *BEND5* methylation in early stages (I and II). *BEND5* was considered hypermethylated when the methylation level relative to the *ACTB* gene exceeded 0.022, which was the 2-fold of medium methylation level of *BEND5* relative to the *ACTB* gene in all patients. (**B**) Correlation between *BEND5* promoter methylation and mRNA expression in the matched normal and tumor tissues was estimated using the Spearman rank correlation (*r*s). (**C**) Colon adenocarcinoma with negative BEND5 expression (original magnification, ×200). (**D**) Colon adenocarcinoma with high BEND5 expression (original magnification, ×200). Scale bars represent 100 µm. (**E**) Kaplan–Meier survival curves were used to compare the overall survival between CRC patients with low and high BEND5 protein expression.

To determine whether the hypermethylation of *BEND5* is associated with mRNA expression, we analyzed BEND5 mRNA expression in 47 paired CRC tissues. In 68% (32/47) of the paired tissues, BEND5 mRNA expression was 2-fold lower in the tumor tissue than in the normal colorectal tissue (Supplementary Figure 2). In contrast to the normal tissues, *BEND5* was significantly hypermethylated, with low mRNA expression in the colorectal tumors (Figure 2A, Spearman rho = −0.352, *P* = 0.0005). In addition, hypermethylation of *BEND5* was associated with a poor prognosis in the stage I and II (Figure 2B, *P* = 0.037). Multivariate Cox proportional-hazards survival analysis were further adjusted by sex, age, tumor type, location, differentiation, stage showed that hypermethylation of *BEND5* genes was significantly and independently associated with overall survival in 105 CRC patients (Table 2, *P* = 0.023).

**Table 2:** Cox proportional-hazards survival analysis in patients with colorectal cancer

	Univariate analysis^a^				Multivariate analysis^a^			
Variable	*n*	HR	95% CI	*P*-value^b^	*n*	HR	95% CI	*P*-value
Sex (female vs. male)	98	1.391	0.549−3.524	0.487	95	1.011	0.316−3.234	0.985
Age (> 65 vs. < 65 years)	98	0.938	0.370−2.377	0.892	95	1.232	0.412−3.681	0.709
Tumor type (other types vs. adeno)	98	2.832	1.003−7.993	0.049^*^	95	2.034	0.226−18.346	0.527
Location (rectal vs. colon)	98	1.221	0.482−3.092	0.673	95	1.165	0.427−3.181	0.766
Differentiation (poor vs. moderate and well)	96	2.588	0.460−14.552	0.280	95	1.457	0.180−11.796	0.724
Tumor stage (later vs. earlier stage)	98	2.181	1.249−3.809	0.006^**^	95	2.728	1.359−5.475	0.005^**^
BEND5c methylation (higher vs. lower methylation)	96	1.213	1.003−1.465	0.046^*^	95	1.278	1.035−1.578	0.023^*^

^a^These results were analyzed by the Cox proportional-hazards survival analysis. For multivariate Cox proportional-hazards survival analysis, the data were adjusted by sex, age, tumor type, location, differentiation and stage. In 95 CRC patients, 17 patients were dead and 78 patients were alive.

^b*^P < 0.05; ^**^P < 0.01.

^c^The BEND5 methylation levels were derived from CRC tumors of 96 patients using QMSP.

To understand the relationships between prognosis and BEND5 protein expression in the CRC patients, immunohistochemical analyses of South Korean CRC tissue microarrays were performed. BEND5 protein expression was weak or absent in 86% (75/87) of the CRC patients (Figure 2C and 2D, Supplementary Table 2). Consistently, patients with no or low BEND5 protein expression exhibited a lower survival rate than those with high BEND5 protein expression (Figure 2E, *P* = 0.037).

### *BEND5* promoter hypermethylation and low mRNA expression in CRC tissues from the TCGA data set

**Figure 3:**
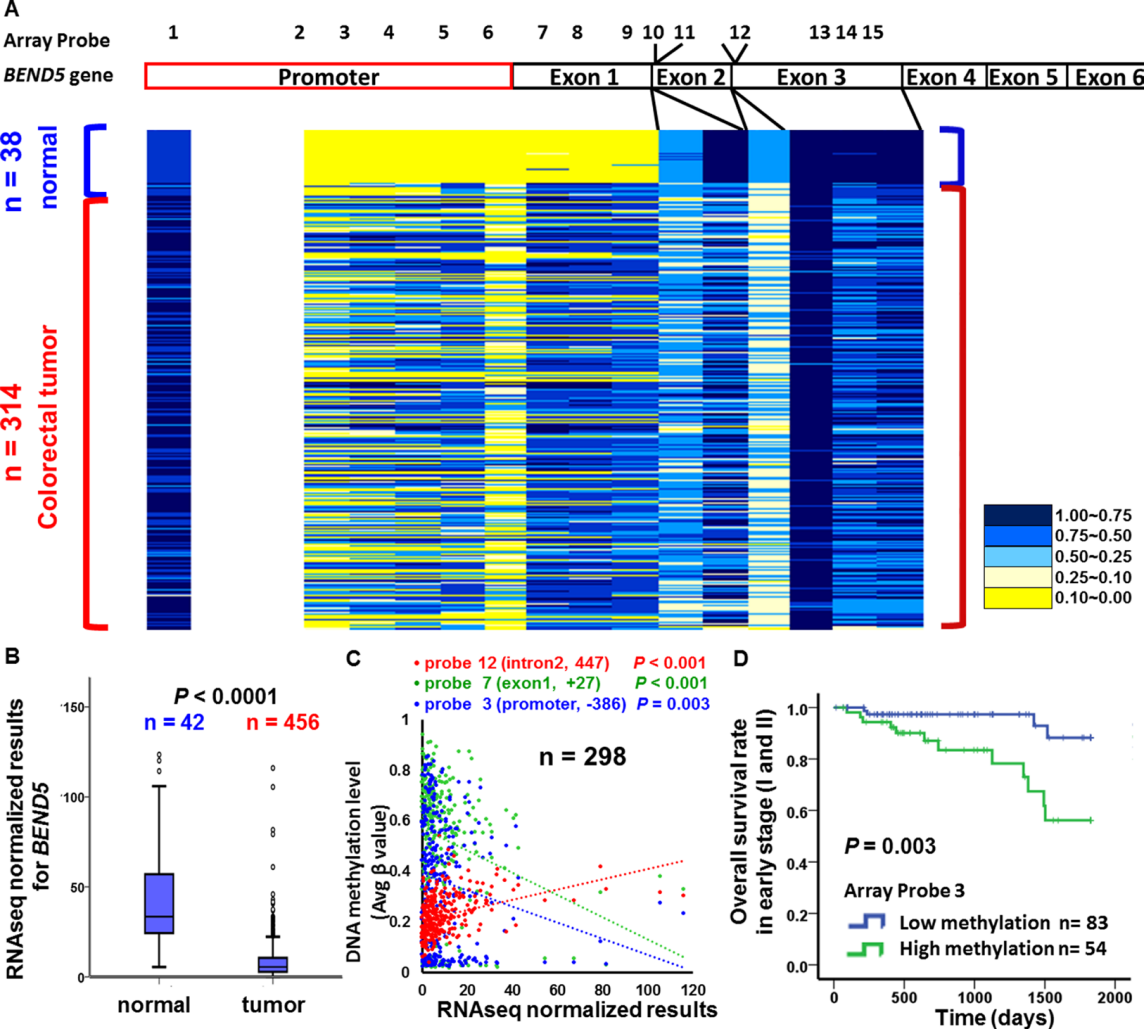
BEND5 DNA methylation and mRNA analysis from the TCGA data set (**A**) Differentially methylated CpG sites on *BEND5* were identified in 314 CRC tumors and 38 normal colorectal tissues by using the Illumina Methylation 450K array-based assay. (**B**) BEND5 was significantly downregulated in CRC tumors compared with normal tissues according to RNA sequencing data for 468 CRC patients. (**C**) Pearson correlation test for tissues from 298 CRC patients in the TCGA data set revealed a correlation between DNA methylation and RNA sequencing. (**D**) Kaplan–Meier survival curves were used to compare the overall survival between CRC patients with low and high *BEND5* methylation in early stages (I and II). *BEND5* was considered hypermethylated at an average β value of > 0.5.

To determine whether the candidate TSG *BEND5* is also altered in CRC patients from other countries, we analyzed the data of Illumina Infinium HumanMethylation450 BeadChip array and RNA sequencing from TCGA data set. Again, the exon 1 region of *BEND5* was hypermethylated in 38 colorectal tumor tissues, but not in the matched normal colorectal tissues (Figure 1B). To confirm this finding, we analyzed data from the TCGA data set. As shown in Figure 3A, the promoter region of *BEND5* was hypermethylated. Notably, hypermethylaion (β > 0.5) was detected in 109 and 216 of the 314 CRC tumors for probe 3 and probe 7 of the promoter and exon 1 sequences, respectively. Analysis of RNA sequencing data from the TCGA showed that BEND5 mRNA expression was markedly significantly reduced in the CRC tumor tissues compared with the matched normal colorectal tissues (Figure 3B, *P* < 0.001). Moreover, the Pearson correlation test revealed a significantly negative correlation between BEND5 mRNA expression and *BEND5* hypermethylation in the promoter −386 region (array probe 3, *P* = 0.003), promoter -211 region (array probe 5, *P* = 0.011) and the exon 1 region +27 (array probe 7, *P* < 0.001), exon 1 region +33 (array probe 8, *P* = 0.012), exon 1 region +187 (array probe 9, *P* = 0.042). By contrast, the correlation was positive in the *BEND5* gene body (intron 2) region (array probe 12, *P* < 0.001) (Figure 3C, *n =*298 tumors; and Supplementary Figure 3, *n =*11 paired tumors and normal tissues). Compared with those exhibiting low methylation, prognosis was significantly poorer in patients exhibiting hypermethylation of the *BEND5* promoter who had stage I or II CRC (Figure 3D, *P* = 0.003). Multivariate Cox proportional hazards regression analysis further showed that hypermethylation of *BEND5* genes was significantly associated with 5-year overall survival (Supplementary Table 3, *P* = 0.033).

### Hypermethylation of *BEND5* leads to low BEND5 mRNA and protein expression

**Figure 4:**
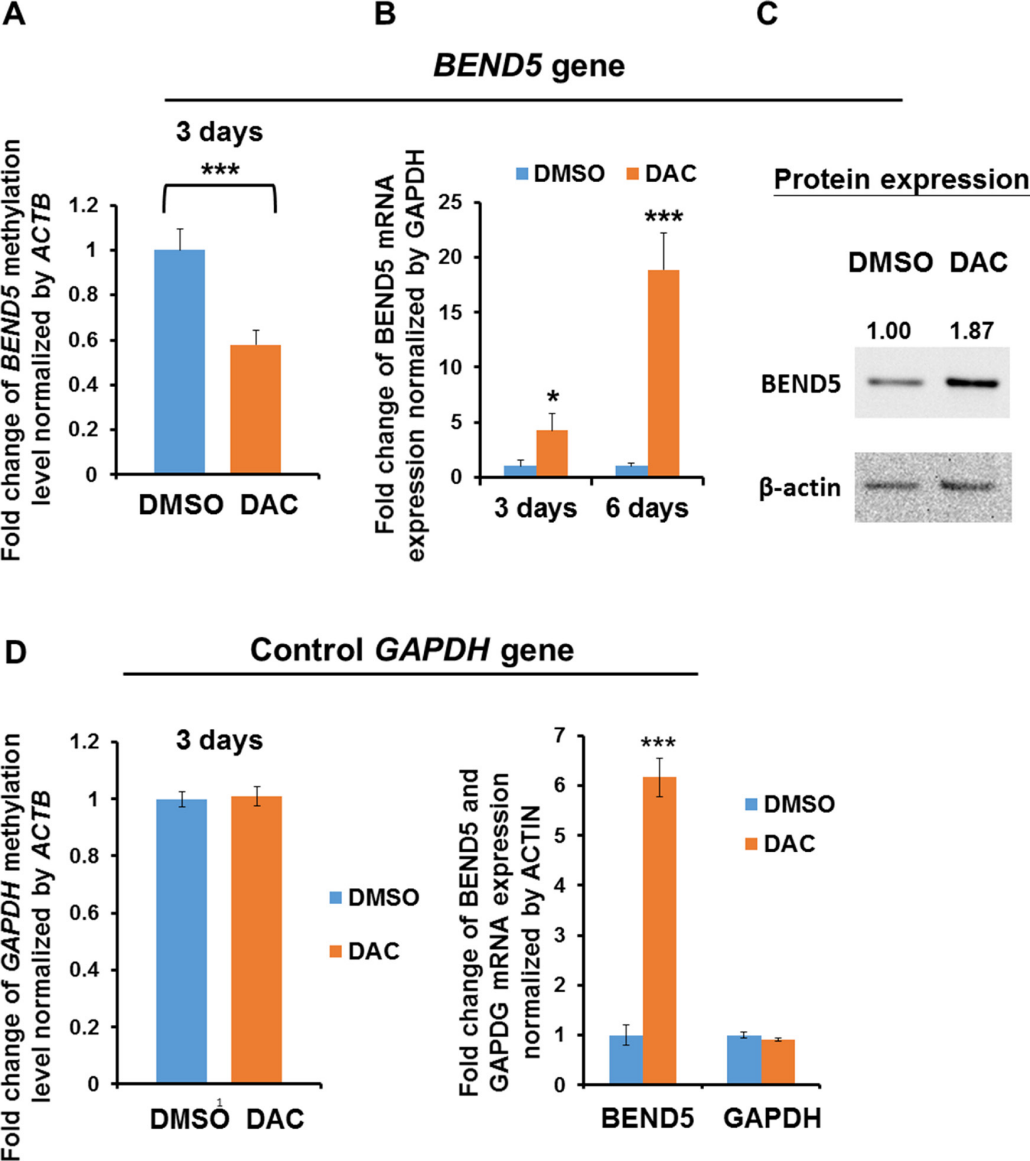
BEND5 expression was modulated by DNA methylation (**A**) Methylation of the *BEND5* promoter decreased after treatment of DLD-1 cells with the DNA-demethylating agent DAC (5 μM) for 72 h. (**B**) BEND5 mRNA and (**C**) BEND5 protein increased following DAC treatment. (**D**) The DNA methylation level and mRNA expression of the control gene *GAPDH* were not changed by DAC treatment. The data are presented as the mean ± SD, ^*^P ≤ 0.05, ^***^P ≤ 0.001. The experiments were performed with at least three technical replicates. The *t* test was used to calculate the group differences in all the experiments.

In the clinical samples of CRC tissues, *BEND5* exhibited low expression and hypermethylation. To investigate whether *BEND5* expression is mainly modulated by hypermethylation, DLD-1 cells were treated with DAC for 3–6 days. The data showed that *BEND5* methylation decreased after 3 days of DAC treatment (Figure 4A), whereas mRNA and protein expression increased after DAC treatment (Figure 4B and 4C), suggesting that *BEND5* promoter hypermethylation is the main mechanism of BEND5 silencing.

### BEND5 protein represses colon cancer cell proliferation

**Figure 5:**
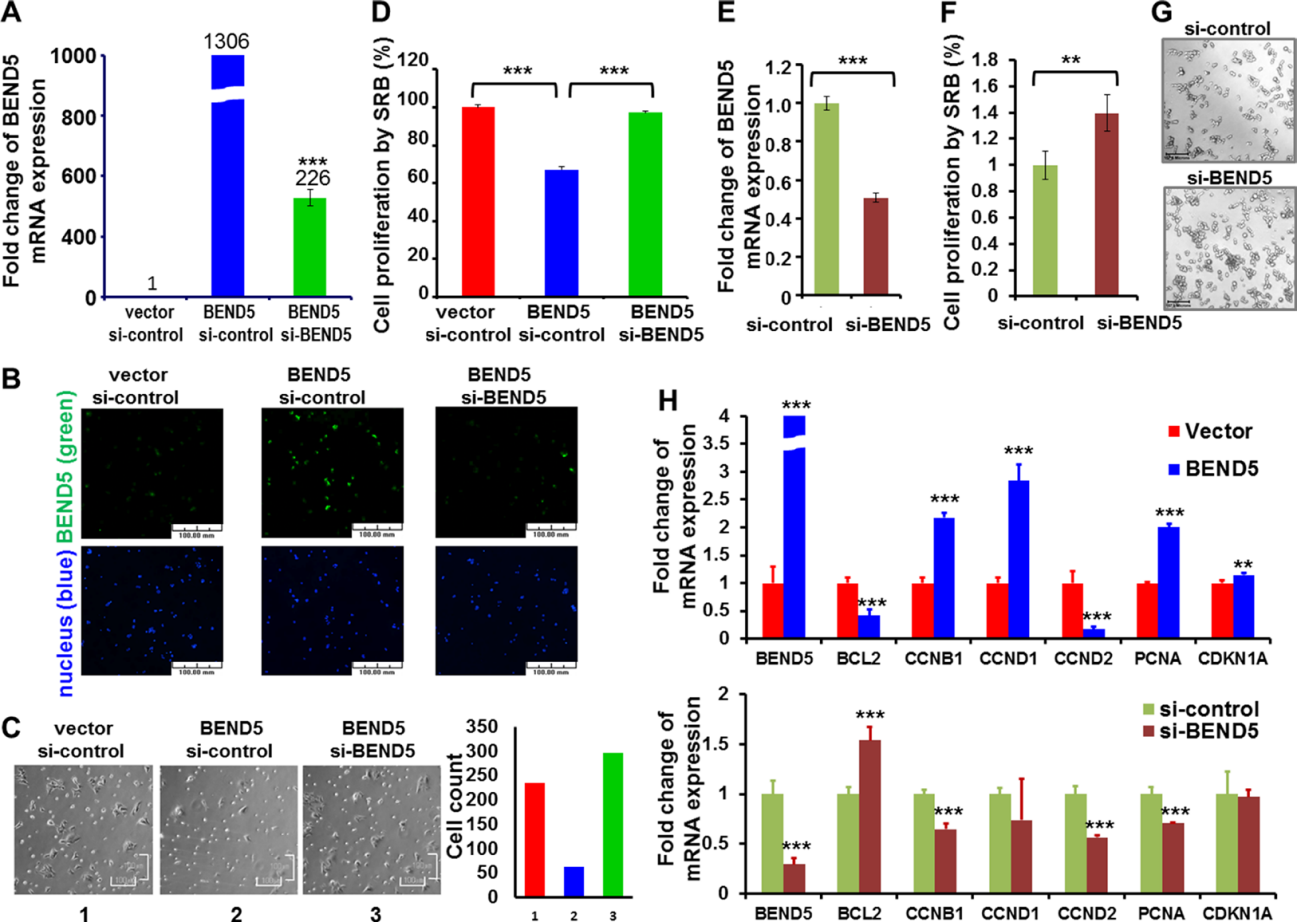
BEND5 may repress cell growth in colon cancer cell *(***A**) Plasmid of *BEND5* and/or si-BEND5 was transfected to DLD-1 cells for 24 h and then analyzed through real-time RT-PCR and immunofluorescence staining for mRNA and (**B**) protein expression, respectively. Scale bars represent 100 µm. (**C**) The bright view was taken for cell morphology. Scale bars represent 100 µm. (**D**) Cells were analyzed using the SRB assay for cell growth. (**E**) Knockdown of BEND5 was performed in COLO 320DM colon cells. Real-time PCR was performed at 48 h; (**F**) Cells were analyzed using the SRB assay for cell growth at 48 h; (**G**) The bright view was taken for evaluating cell morphology. Scale bars represent 157.5 µm. (**H**) Real-time RT-PCR was performed to analyze the expression of *CCNB1*, *PCNA*, *CCND1*, and *BCL2* after *BEND5* overexpression in DLD-1 colon cancer cells or *BEND5* knockdown in COLO 320 DM colon cancer cells. The data are presented as means ± SD, ^**^P ≤ 0.01, ^***^P ≤ 0.001. The *t* test was used to calculate the group differences in all the experiments. The experiments were performed with at least two biological duplicates and three technical replicates. Panels B and C were not taken in the same fields.

To study the biological roles of BEND5 protein in CRC cells, overexpression or knockdown of *BEND5* was achieved in DLD-1 cells by electroporation. The gene manipulation efficiency was determined through real-time RT-PCR and immunofluorescent staining (Figure 5A and 5B). Microscopic observation showed that *BEND5* overexpression repressed the growth of DLD-1 cells, as compared with that of a vector control or the BEND5 knockdown group (Figure 5C). According to the cell proliferation SRB assay, BEND5 also repressed DLD-1 cancer cell growth by 32.9% (Figure 5D), whereas si-BEND5 knockdown increased CRC cell proliferation (Supplementary Figure 4). To verify whether the decrease in BEND5 expression induces cell growth, knockdown of *BEND5* gene expression was achieved in COLO 320DM human colon cancer cells, which exhibit higher endogenous BEND5 expression (Supplementary Figure 5). BEND5 knockdown increased COLO 320DM colon cancer cell growth by 39.75% (Figure 5E).

We also determined whether BEND5 directly or indirectly regulates cell growth-related control genes that contain BEND5 binding sites in their promoter regions, such as *CCND1*, *CCND2*, *CCNB1*, *CDKN1A*, *PCNA*, and *BCL2* (Supplementary Table 4). *BEND5* overexpression in DLD-1 colon cancer cells and *BEND5* knockdown in COLO 320 DM colon cancer cells indicated that BEND5 could significantly change their expression levels (Figure 5E), suggesting that BEND5 may directly or indirectly regulate *CCNB1*, *PCNA* and *BCL2*.

## DISCUSSION

Aberrant promoter hypermethylation of CpG islands associated with TSGs can cause transcriptional silencing, contributing to tumorigenesis. In this study, using Illumina Infinium HumanMethylation450 BeadChip arrays, we discovered that highly methylated CpG sites in the promoter and exon 1 regions of *BEND5* occurred in the CRC tumor tissues, but not in the corresponding normal colorectal tissues. Furthermore, QMSP confirmed *BEND5* hypermethylation in the CRC tumor tissues compared with the normal tissues. In the Asian cohorts, earlier-stage CRC patients without metastasis having hypermethylation of the promoter region of *BEND5* in their tumors had a poor prognosis. The TCGA data set provided similar results for the Caucasian cohort. Moreover, patients with low BEND5 protein expression also had a poor prognosis.

BEND5 belongs to the BEN domain family, the members of which are found in several animal proteins. Previous studies have suggested that the BEN domain mediates protein–DNA and protein–protein interactions during chromatin organization and transcription [18, 19, 21]. BANP (also referred to as BEND1 or SMAR1) was the first BEN domain member discovered [22]. BANP reportedly binds to the AT-rich region and mediates *CCND1* repression [23]. Functional data are presented in this study to demonstrate, for the first time, that BEND5 can reduce cancer cell survival (Figure 5). Previous studies have suggested that BEND5 binds to the TCCAATTGGA sequence or the TCYAATHRGAA sequence and regulates transcription [18]. To survey the sequence on the promoter regions of cell growth-related control genes, we found similar BEND5 binding sequences in the *BCL2*, *CCND1*, *CCND2*, *PCNA*, *CDKN1A*, and *CCNB1* promoter regions (Supplementary Table 2). *BEND5* overexpression and knockdown analysis indicated that BEND5 may directly or indirectly regulate *CCNB1*, *PCNA*, *CCND1*, and *BCL2* (Figure 5). CCND1, PCNA, CCNB1, and BCL2 are involved in cell cycles, cell survival, and cell proliferation control [24–26]. PCNA is also involved in DNA repair and exerts its functions through interaction with various proteins involved in DNA synthesis, repair, and recombination [27]. The most obvious change in expression is *BL2*. Further research is warranted to determine whether alterations of BEND5 in CRC cells contribute to tumorigenesis by deregulating the expression of *BCL2*.

In addition to CRC tissues, this study detected low BEND5 expression in several CRC cell lines, compared with colon tissues from normal controls (Supplementary Figure 5). Through the *in vitro* cell model, both the knockdown and overexpression experiments support that BEND5 can repress cell proliferation (Figure 5 and Supplementary Figure 4), indicating that BEND5 may be a candidate TSG. Flow cytometry revealed that BEND5 knockdown induced a slight decrease in the proportion of cells in the G1 phase and a slight increase in that in the S phase (Supplementary Figure 6). Notably, in the G1 phase, BEND5 reduced the proportion of DLD-1 cancer cells by 32.9% but increased cell cycle arrest by only 5% (Figure 5D and Supplementary Figure 6). These data suggest that BEND5 reduces the cell proportion through other mechanisms such as apoptosis, autophagy, and cellular differentiation, rather than cell cycle arrest.

The current study on Taiwan CRC samples (Table 1) characterized, in detail, the methylation of the *BEND5* promoter and exon regions (Figure 1 and Supplementary Figure 1). Similar results were previously reported for 24 CRC patients in Bangladesh [28]. Our analysis of the TCGA data set also revealed that *BEND5* was hypermethylated, leading to low mRNA expression. In addition, although the overall epigenetic pattern of promoter hypermethylation and gene body demethylation in the BEND5 sequences was similar between the CRC tissues of the Asian and Caucasian cohorts, the CRC tumor tissues from the TCGA data set displayed even lower methylation levels in the promoter region (probes 2–4 and 6). Furthermore, the normal colorectal tissues from the TCGA data set exhibited higher methylation levels at the intron 1 position 3710 site (probe 11) (Figure 1).

DNA methylation of the promoters and the exon 1 region results in has a well-known gene silencing; however, a positive correlation between gene body methylation and expression was reported recently [29]. Our study revealed that hypermethylation of the promoter and exon 1 regions correlated negatively with RNA expression (e.g., probes 3 and 7), whereas BEND5 gene body methylation correlated positively with RNA expression (e.g., probe 12) (Figure 3 and Supplementary Figure 3).

In our case collection, BEND5 mRNA expression in more than two-thirds of our CRC tumor tissues (32/47) was less than 50% of that in the normal control colorectal tissues, as detected by real-time PCR. By contrast, 34/41 (82.9%) of the tumor samples from the TCGA data set exhibited a significant reduction in the BEND5 transcript level, as demonstrated by RNA sequencing (Supplementary Figure 2). This disparity might be attributable to differences in ethnicity or methodology. Taken together, patients with low BEND5 protein expression had a much poorer survival rate than those with high BEND5 protein expression (Figure 2), indicating that low mRNA or protein expression of BEND5 in colorectal tumors is prevalent and is clinically significant for both Asian and Western countries. Decitabine (DAC) treatment increased BEND5 mRNA and protein expression through *BEND5* promoter demethylation (Figure 4). Although the protein expression only increase by 1.87 fold. We suggested that this finding is probably because of the short half-life of the BEND5 protein or some other protein modification, which resulted in changes in the protein half-life or protein expression. The combination of DAC with gefitinib has been found to exert synergistic anticancer activity in colon cancer cells [30]. In addition, DAC sensitizes colorectal cancer cells to topoisomerase inhibitors (irinotecan, etoposide, doxorubicin, and mitoxantrone) [31]. Whether DAC exerts relevant anticancer effects through BEND5 expression induction is worthy of further investigation. In the current study, BEND5 overexpression could repress colorectal cancer cell proliferation (Figure 5); thus, additional studies should discover drugs that induce BEND5 expression through a Connectivity Map, which uses gene expression signatures to connect small molecules, genes, and disease [32].

Advancements in detection technology have reduced CRC death rates in several Western countries [33]. Therefore, developing biomarkers for early detection and intervention can improve patient outcomes. Recent studies have reported that several TSGs are often methylated in the multistep oncogenesis process from normal colonic epithelium to adenocarcinoma [34]. Comprehensive research on methylated DNA markers, such as *SEPT9*, and the combined analysis of several genes rather than a single gene can improve clinical efficacy of CRC management [34, 35]. In the present study, the *BEND5* promoter and exon 1 regions were hypermethylated only in the CRC tumors, but not in the matched colon tissues (Figures 1–3), suggesting that *BEND5* hypermethylation may be a biomarker of CRC. Because prognosis is poor in earlier-stage CRC patients without metastasis exhibiting hypermethylation of the promoter region of *BEND5* in their tumors (Figures 2 and 3), *BEND5* hypermethylation may be a prognostic indicator that enables identifying high-risk patients for frequent monitoring. Thus, whether detecting *BEND5* hypermethylation in the blood or stool samples of CRC patients can be applied as a noninvasive analytical method for early detection that is worthy of further investigation. In conclusion, functional evaluation of the BEND5 targets and further investigation on BEND5-interacting genes are warranted to develop a useful panel for clinical applications.

## MATERIALS AND METHODS

### Patients and tissue collection

For methylation array analysis, a total of 26 colorectal cancer patients who underwent surgery at Taipei Veterans General Hospital or Taipei Medical University were enrolled, comprising 10 patients with microsatellite-stable tumors, 13 patients with microsatellite instability (MSI)-high tumors, and 3 patients with tumors of unknown microsatellite status. In the MSI-high tumors, no mutation in MMR genes including *MLH1, MSH2, MSH6,* and *PMS2* was detected. For analysis of *BEND5* methylation, 135 CRC patients who underwent surgery at Taipei Veterans General Hospital or Taipei Medical University were enrolled. Before clinical data and sample collection, written informed consent was obtained from all patients. Patients undergoing preoperative chemoradiotherapy or an emergent operative procedure, those who died within 30 postoperative days, or those with evidence of familial adenomatous polyposis or Lynch syndrome were excluded from this study. Frozen human tissue samples were obtained from the Taipei Medical University (TMU) Joint Biobank and Taipei Veterans General Hospital Biobank. Sections of cancerous tissue and corresponding noncancerous tissues were reviewed by a senior gastrointestinal pathologist. Clinical data on sex, personal and family medical history, tumor location, TNM tumor stage, tumor differentiation, MSI, pathological features, and follow-up conditions, which were prospectively collected, were obtained from the TMU Joint Biobank and Taipei Veterans General Hospital Biobank.

Following surgery, patients were monitored every 3 months for the first 2 years and semi-annually thereafter. The follow-up protocol included physical examination, digital rectal examination, carcinoembryonic antigen analysis, chest radiography, abdominal sonogram, and computerized tomography, if required. Proton emission tomography or magnetic resonance imaging was arranged for patients with an elevated carcinoembryonic antigen level but tumor recurrence at an uncertain site.

### DNA and RNA extraction

Genomic DNA from matched pairs of primary tumors and adjacent colorectal tissues from the same patient was prepared using the QIAamp DNA Mini Kit (Qiagen, Bonn, Germany, Cat. No. 51306). For RNA extraction, tumor and normal specimens were frozen immediately after surgical resection and stored at −80 °C. Total mRNA was extracted using the RNeasy Plus Mini Kit (Qiagen, Bonn, Germany, Cat. No. 74134). After DNA and RNA quantification, the purity was verified by measuring the A260/A280 ratio (which ranged from 1.8 to 2.0). cDNA was synthesized using the iScript cDNA Synthesis Kit (Bio-Rad Laboratories, Shanghai, China, Cat. No. 170–8891) according to the manufacturer’s instructions.

### Genome-wide methylation analysis

The genome-wide methylation analysis of 26 paired CRC tissues and corresponding noncancerous colon tissues was performed using the Illumina Infinium HumanMethylation450 BeadChip array (Illumina, San Diego), as previously reported [16]. The array contains more than 450 000 methylation sites and provides genome-wide coverage of the gene region and CpG island coverage, including 99% of Refseq genes. Bisulfite conversion was performed for 500 ng of genomic DNA by using the EpiTect Fast DNA Bisulfite Kit (Qiagen, Bonn, Germany, Cat. No. 59826), according to the manufacturer’s instructions. Methylation scores for each CpG site were represented as “beta” values ranging from 0 (unmethylated) to 1 (fully methylated) by determining the ratios of the methylated signal intensities to the sums of the methylated and unmethylated signal outputs.

### Reverse transcription PCR

To measure BEND5 mRNA expression, real-time reverse transcription PCR (RT-PCR) was performed with the LightCycler 480 (Roche Applied Science, Mannheim, Germany). Real-time PCR was performed using the LightCycler 480 Probe Master Kit (Roche Applied Science, Indianapolis, Indiana, USA, Cat. No. 04707494001) with the specific primers and the corresponding Universal Probe Library probe (Roche Applied Science, USA), according to the manufacturer’s instructions. The glyceraldehyde 3-phosphate dehydrogenase gene (*GAPDH*) was used as a reference gene. The normalized gene expression values obtained using LightCycler Relative Quantification software (Version 2.0, Roche Applied Science) were then compared with those of the control group. BEND5 mRNA expression was considered low if the mRNA expression level of *BEND5* relative to *GAPDH* was 0.5-fold lower in the colorectal tumor tissue than in the paired normal colorectal tissue. Supplementary Table 5 lists the primers.

### TaqMan quantitative methylation-specific PCR

After bisulfite conversion of genomic DNA by using the EpiTect Fast DNA Bisulfite Kit (Qiagen, Bonn, Germany, Cat. No. 59826) according to the manufacturer’s recommended protocol, the DNA methylation level of *BEND5* was measured using TaqMan quantitative methylation-specific PCR (QMSP) by using the LightCycler 480 (Roche Applied Science). QMSP was performed using the SensiFAST™ Probe No-ROX Kit (Bioline, London, UK, Cat. No. BIO-86020) with the specific primers and methyl-TaqMan probe of *BEND5*. Normalized DNA methylation values, which were calibrated to the control group, were obtained using LightCycler Relative Quantification software (Version 2.0, Roche Applied Science).

The *ACTB* gene was used as a reference gene. *BEND5* was considered hypermethylated when the methylation level of *BEND5* relative to that of the *ACTB* gene was at least 2-fold higher in the colorectal tumor compared with the paired normal colorectal tissue sample. The specificity of *BEND5* methylation end products was confirmed by bisulfite sequencing (Supplementary Figure 1A and 1B). Supplementary Table 5 presents the primers.

### Cell line, cell culture, and drug treatment

DLD-1, COLO 320DM, and T84 CRC cell lines, which were obtained from the Bioresource Collection and Research Center (http://www.bcrc.firdi.org.tw/), were cultured in UltraCulture Serum-free medium (Lonza, Walkersville, Maryland, USA, Cat. No. 12–725F) and RPMI1640 (Invitrogen, Grand island, Nebraska, USA). For the demethylation assay of *BEND5*, the DLD-1 cells were treated with Dimethyl sulfoxide (DMSO) or the demethylation agent decitabine (DAC, Sigma-Aldrich, St. Louis, Missouri, USA, Cat. No. SLBN2574V). DAC treatment is efficacious for epithelial tumor cells and is accompanied by decreases in genome-wide promoter DNA methylation and gene re-expression [36]. After treatment, DNA, RNA, and protein were extracted, and methylation and expression levels were analyzed. DAC was dissolved in DMSO.

### Immunoblot analyses

For Western blotting, the cells were lysed on ice in radioimmunoprecipitation buffer (0.05 M Tris-HCl [pH 7.4], 0.15 M NaCl, 0.25% deoxycholic acid, 1% Igepal CA-630, and 1 mM ethylenediaminetetraacetic acid). The lysates were centrifuged at 13000 rpm at 4 °C for 10 min. The protein extracts were solubilized in SDS gel loading buffer (60 mmol/L Tris base, 2% SDS, 10% glycerol, and 5% β-mercaptoethanol). Samples containing equal amounts of protein (40 μg) were separated on 8% SDS-PAGE gel by using electrophoresis and electroblotted onto Immobilon-P membranes (Millipore, Bedford, Massachusetts, USA) in transfer buffer. Immunoblotting was performed using antibodies against BEND5 (1:1000, Sigma-Aldrich, SAB2700049, Taiwan, ROC). The *ACTB* gene (1:5000, GeneTex, GTX26276, Texas, USA) was used as an internal control.

### Immunofluorescence staining assay

The cells were seeded in four-well glass chamber slides (Nunc, Bedford, Massachusetts, USA). After transfection for 24 h, the cells were fixed in 4% formaldehyde and stained with 4’,6-diamidino-2-phenylindole (DAPI) and anti-BEND5 antibody (1:1000, Sigma-Aldrich, SAB2700049, Taiwan, ROC). Images were captured using the Olympus IX71 Inverted Microscope System (Olympus America, Center Valley, PA, USA).

### Immunohistochemistry assay

Two sets of tissue microarrays of CRC were purchased from SuperBioChips Laboratories (catalog numbers: CD4 and CDA3; Seoul, South Korea). The two sets of tissue microarrays were composed of well-preserved colorectal tumor tissues obtained from 87 South Korean cases of CRC and were used for the immunohistochemical evaluation of BEND5 expression. The pathologic diagnoses of these cases were microscopically confirmed. Immunohistochemical staining was performed using an iView DAB detection kit (Ventana, Tucson, Arizona, USA) on a BenchMark XT autostainer. The sections were incubated with BEND5 antibody (1:100, Sigma-Aldrich, SAB2700049, ROC) for 1 h at 37 °C. This assay included both positive and negative controls. The researchers who evaluated the immunohistochemistry staining results were blinded to the clinical follow-up data. The intensity of BEND5 expression was identified semiquantitatively as no expression, low expression (weaker than or equal to the expression intensity of normal colon epithelium), or high expression (stronger than the expression intensity of normal colon epithelium).

### cDNA expression construct, RNAi, and transfection

The expression plasmid of *BEND5* was obtained from OriGene (Rockville, Maryland, USA). *BEND5* interference RNA (RNAi) was obtained from Life Technology (Cat. No. s36022, Carlsbad, California, USA). Transfections were performed in DLD-1 and COLO 320DM colon cancer cells using the Neon Transfection System 10 μL Kit, according to the manufacturer’s protocol.

### Cell cycle distribution assay

Cell cycle distribution was determined through flow cytometry. The DLD-1 cells (1 × 10^6^) were trypsinized and fixed overnight with 80% ethanol at −20 °C. The fixed cells were stained with a solution containing 20 μg/mL propidium iodide, 200 μg/mL RNase A, and 0.1% Triton X-100 for 30 min in the dark. Cell cycle distributions were analyzed using the FACSCanto II flow cytometer (BD Biosciences, San Jose, California, USA), and calculations were performed using ModFIT LT Version 2.0 software (Verity Software House).

### Sulforhodamine B assay

A sulforhodamine B (SRB) assay was used to determine the cell growth rate. The DLD-1 cells were seeded in 96-well plates at a density of 8000 cells/well and incubated for 24 h. The cells were fixed with 10% trichloroacetic acid for 10 min. After staining with SRB for 30 min, excess dye was removed by washing the cells five times with 1% acetic acid. Cell growth was assessed using a microplate reader to determine the absorbance of the SRB solution at 515 nm. Growth inhibition rates were calculated as follows: cell growth inhibition rate (%) = 100 − [(Ti − Tz)/(C − Tz)] × 100 (Ti ≥ Tz), where Ti = OD of the inhibitor sample, Tz = OD of basal cells, and C = OD of the control. The images were acquired using an inverted microscope (EVOS, AMG, USA) at the indicated time points. The cell counting was measured and analyzed using Image J software.

### The cancer genome atlas data analysis

The results of the Western cohort are, on the whole, based on data generated by The Cancer Genome Atlas (TCGA) Research Network (http://cancergenome.nih.gov/).

### Statistical analyses

All statistical analyses were performed using SPSS (SPSS Inc., Chicago, Illinois, USA). The Fisher’s exact test was used to compare the CRC patients in terms of *BEND5* methylation, protein and other clinical data, including age, sex, tumor type, TNM tumor stage, vascular invasion, differentiation grade, location, and MSI status. Pearson correlation and Spearman correlation were used to analyze the correlation between the DNA methylation and mRNA expression of *BEND5*. An independent *t* test was used to determine whether BEND5 mRNA expression differed between the cells from normal tissues and those from colorectal tumors. The *t* test was also used to compare cells transfected with or without BEND5 and those with or without drug treatment. The overall survival curves were calculated using the Kaplan–Meier method, and comparisons were performed using the log-rank test. We used Cox proportional hazards survival analysis to estimate the role of *BEND5* methylation levels in the poor survival time of Taiwanese and TCGA CRC patients. Patients who were alive until the end of follow-up or those who died during the follow-up period were recorded. Multivariate Cox proportional hazards survival analysis was further adjusted for age, sex, tumor subtype, tumor location, differentiation grade, and tumor stage to evaluate the independent role of *BEND5* hypermethylation in the overall survival of CRC patients.

## SUPPLEMENTARY MATERIALS FIGURES AND TABLES


