# Supplementary material for: Hypermethylation of BEND5 contributes to cell proliferation and is a prognostic marker of colorectal cancer
Source: Oncotarget. 2017 Nov 1;8(69):113431–43. doi: 10.18632/oncotarget.22266 (PMC5768337; doi:10.18632/oncotarget.22266)
Supplement: Supplementary file 1 [file oncotarget-08-113431-s001.pdf]

Hypermethylation of *BEND5* contributes to cell proliferation and is a prognostic marker of colorectal cancer

SUPPLEMENTARY MATERIALS

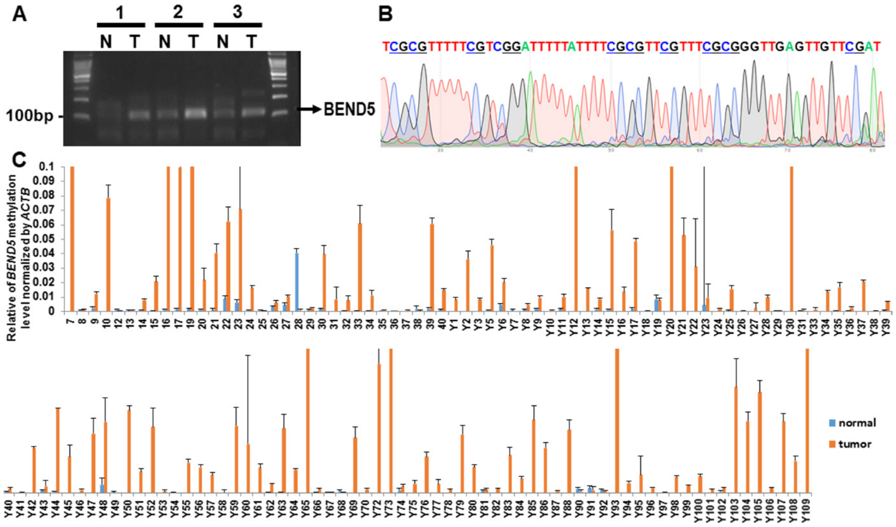

Supplementary Figure 1: Hypermethylation of the *BEND5* promoter gene was analyzed in CRC patients. (**A**) The end products of BEND5 QMSP were run in the gel shown on the right molecular weight. (**B**) Bisulfite sequencing was performed to verify the specificity of the BEND5 QMSP assay. (**C**) Raw data for the BEND5 QMSP assay in 133 CRC patients. The data are presented as means ± s.d. The experiments were performed with at least three technical replicates.

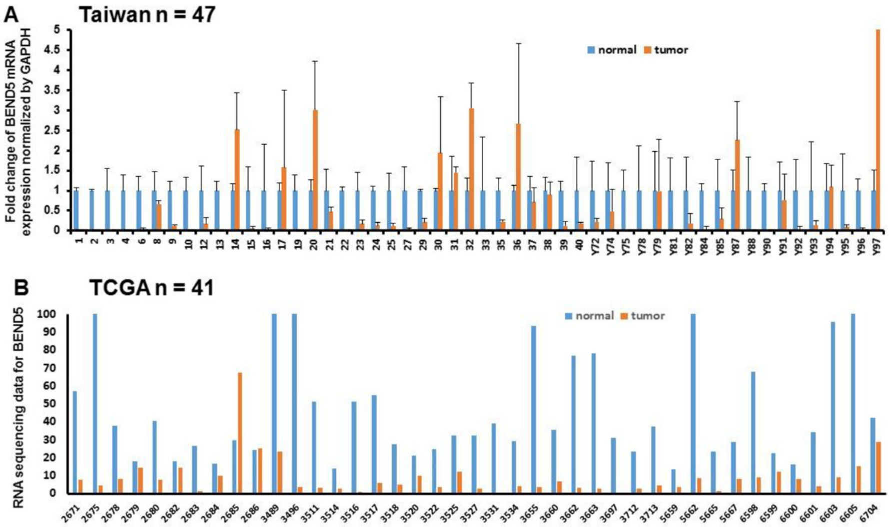

Supplementary Figure 2: The BEND5 mRNA expression levels were analyzed in CRC tumors and paired normal colorectal tissues. (**A**) The BEND5 mRNA expression levels were analyzed using real-time RT-PCR in 47 patients in Taiwan. The data are presented as means ± s.d. The experiments were performed with at least three technical replicates. (**B**) The BEND5 mRNA expression levels were analyzed through the RNA sequencing of paired tissues from normal controls and 41 paired CRC patients from TCGA.

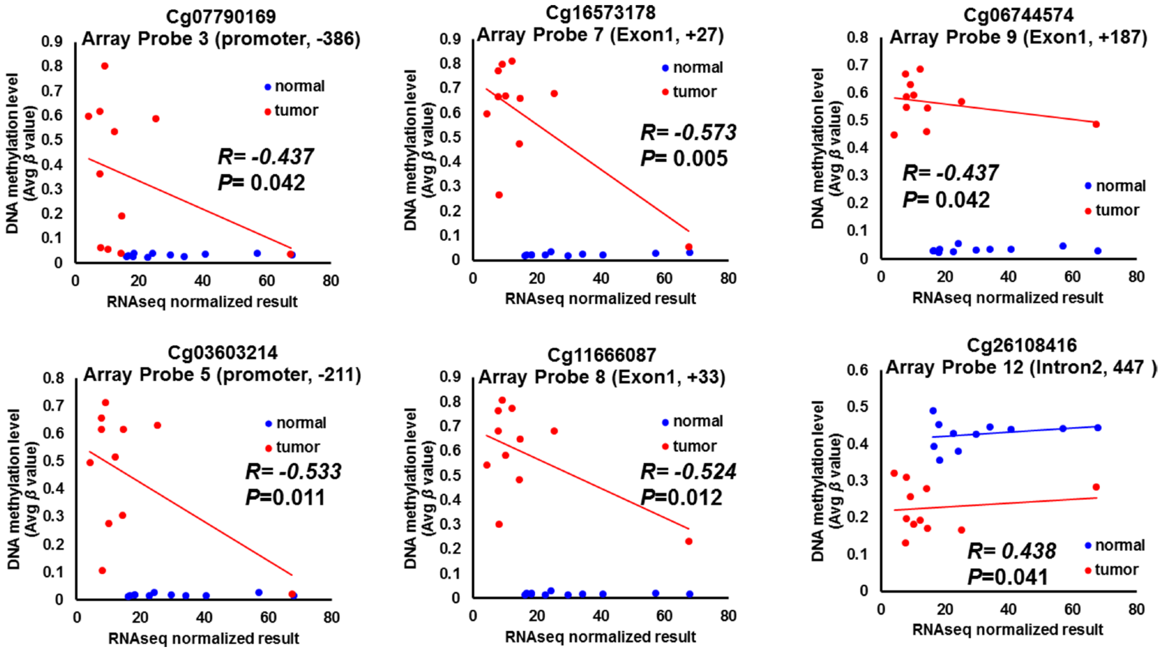

Supplementary Figure 3: Pearson correlation analysis of tissues between DNA methylation and RNA sequencing in 11 normal controls and 11 paired CRC patients.

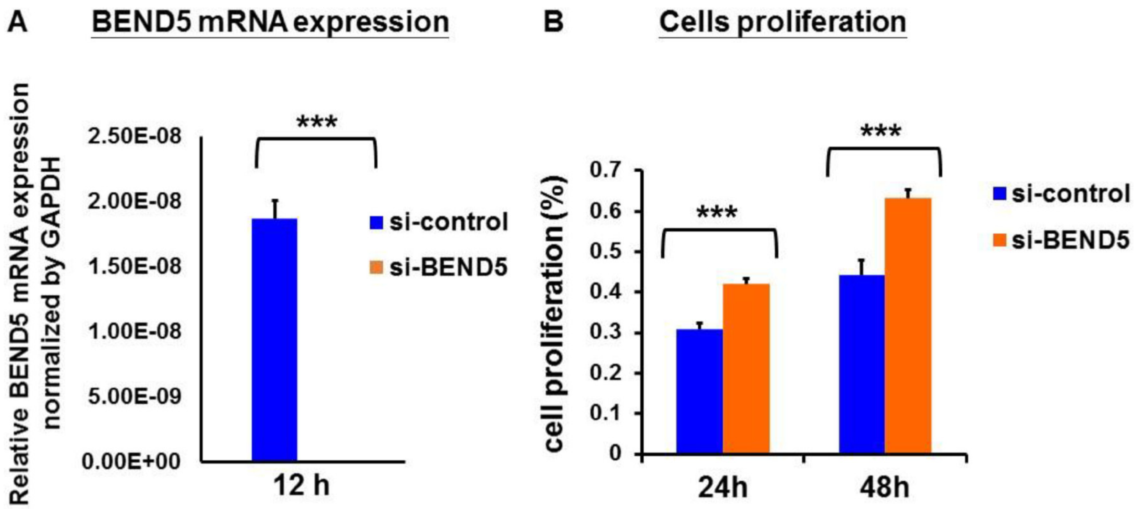

Supplementary Figure 4: Knockdown of endogenous BEND5 mRNA expression induced proliferation of DLD-1 CRC cells. (**A**) BEND5 mRNA expression analyzed using real-time RT-PCR. (**B**) Cell proliferation was analyzed using the SRB assay. The data are presented as means ± s.d., $^{***}P \leq 0.001$. The experiments were performed with at least three technical replicates.

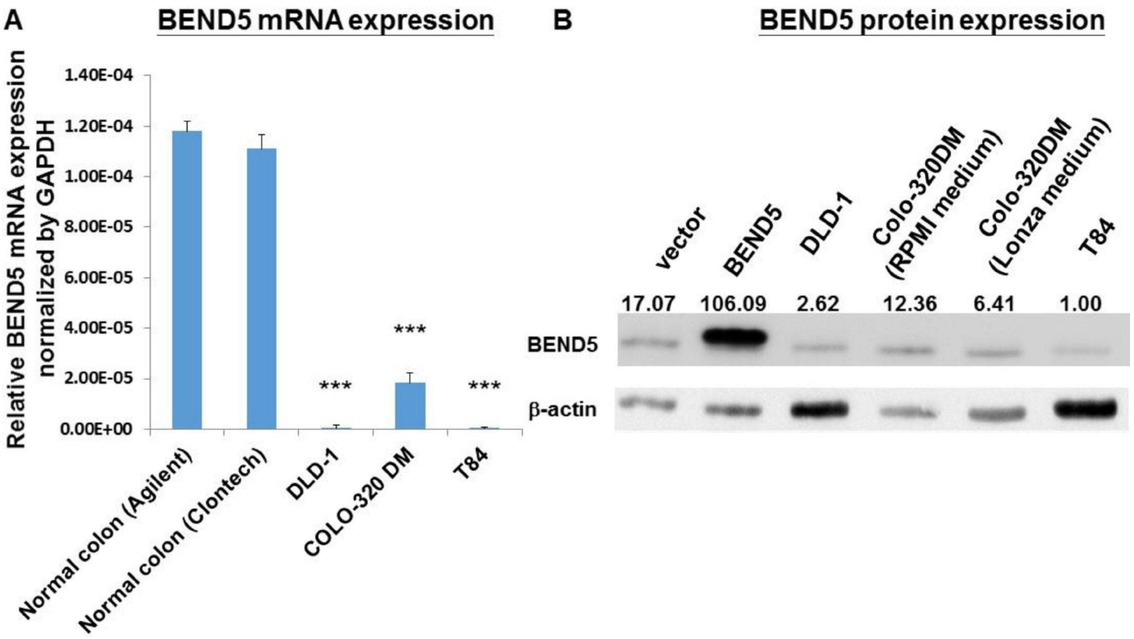

Supplementary Figure 5: BEND5 mRNA and protein expression analysis in normal tissue and cells. (**A**) BEND5 mRNA was highly expressed in normal colon tissues but was not expressed in CRC cell lines. The data are presented as means ± s.d., $^{***}P \leq 0.001$. The experiments were performed with at least three technical replicates. (**B**) The BEND5 protein expression annualized by Western Blot in CRC cell lines and BEND5 plasmid transfected DLD-1 cell lines.

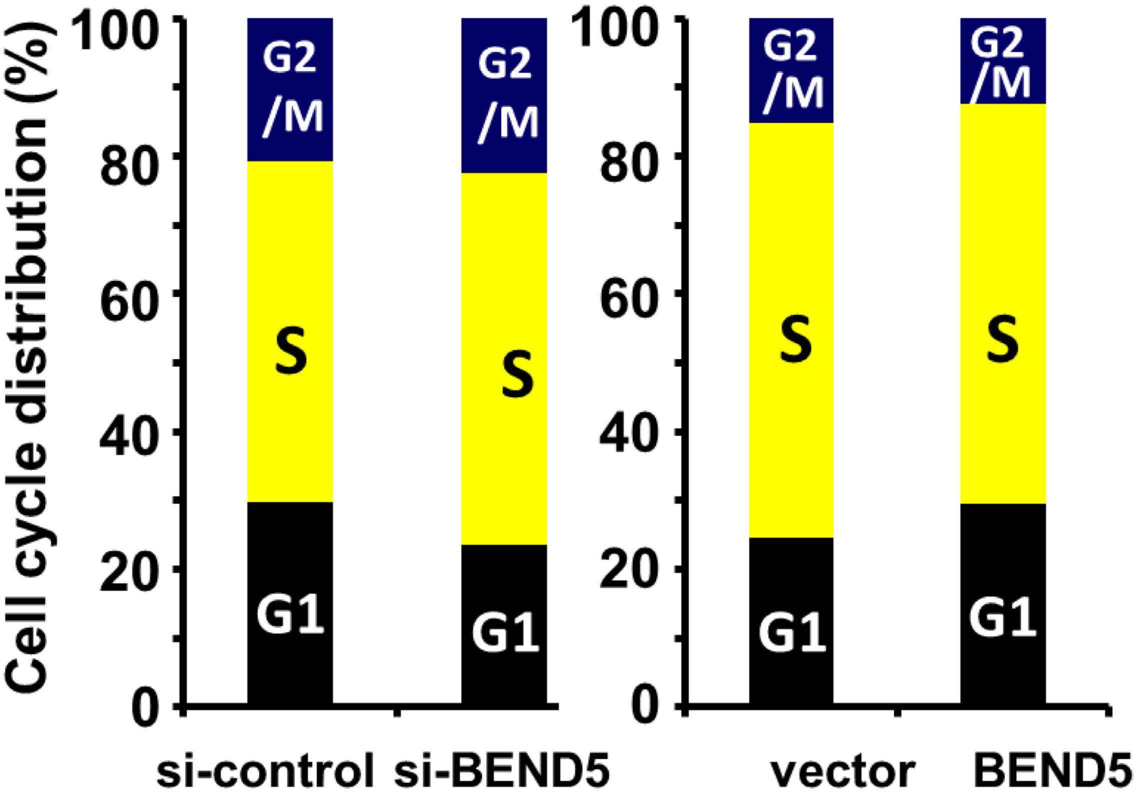

Supplementary Figure 6: Cells were analyzed using flow cytometry for cell cycle distribution.

Supplementary Table 1: The clinical parameters of 26 paired CRC patients who analyzed by Illumina Infinium HumanMethylation450 BeadChip array assays

Characteristics		Total
		***n* (%)**
**Total**		26
**Clinicopathological parameters**		
**Age**	< 60	8 (30.8)
	> 60	18 (69.2)
**Sex**	Male	18 (69.2)
	Female	8 (30.8)
**Tumor type**		
Adeno		21 (84.0)
Mucinous		1 (4.0)
Others		3 (12.0)
**Tumor stage**		
Early (stage I and II)		16 (61.5)
Late (stage III and IV)		10 (38.5)
**Primary tumor**		
Submucosa		1 (3.8)
Muscularis propria		5 (19.2)
Subserosa		17 (65.4)
Penetrate the visceral		3 (11.5)
**Regional lymph nodes**		
No regional lymph node metastasis		17 (65.4)
Metastasis in regional lymph nodes		9 (34.6)
**Distant metastasis**		
No distant metastasis		24 (92.3)
Distant metastasis		2 (7.7)
**Differentiation grade**		
Well		3 (11.5)
Moderate		18 (69.2)
Poor		5 (19.2)
**Location**		
colon		20 (76.9)
Rectal		6 (23.1)

Supplementary Table 2: BEND5 protein expression in relation to clinical parameters for CRC[a]

Characteristics		Total	BEND5 protein		
			Low	High	*P*-value
		n[b]	*n* (%)	*n* (%)	
**Overall**		94	82 (87.2)	12 (12.8)	
**Clinicopathological parameters**					
**Age**	< 65	66	58 (87.9)	8 (12.1)	0.740
	> 65	27	23 (85.2)	4 (14.8)	
**Sex**	Male	68	58 (85.3)	10 (14.7)	0.500
	Female	26	24 (92.3)	2 (7.7)	
**Tumor type**					
Adeno		83	71 (85.5)	12 (14.5)	0.402
Mucinous		10	10 (100.0)	0 (0.0)	
Small cell		1	1 (100.0)	0 (0.0)	
**Tumor location**					
Colon		74	63 (85.1)	11 (14.9)	0.450
Rectal		20	19 (95.0)	1 (5.0)	
**Differentiation grade**					
Well		19	17 (89.5)	2 (10.5)	0.845
Moderate		57	48 (84.2)	9 (15.8)	
Poor		6	5 (83.3)	1 (16.7)	
**Tumor stage**					
I		3	3 (100.0)	0 (0.0)	0.407
II		42	34 (81.0)	8 (19.0)	
III		34	31 (91.2)	3 (8.8)	
IV		15	14 (93.3)	1 (6.7)	
**Primary tumor**					
Submucosa		0	0 (0.0)	0 (0.0)	0.417
Muscularis propria		7	6 (85.7)	1 (14.3)	
Subserosa		80	71 (88.8)	9 (11.3)	
Penetrate the visceral		7	5 (71.4)	2 (28.6)	
**Regional lymph nodes**					
No regional lymph node metastasis		50	41 (82.0)	9 (18.0)	0.130
Metastasis in regional lymph nodes		44	41 (93.2)	2 (6.8)	
**Distant metastasis**					
No distant metastasis		79	68 (86.1)	10 (13.9)	0.683
Distant metastasis		15	14 (93.3)	1 (6.7)	

[a]These results were analyzed by the Fisher's exact test.
[b]For some categories, the number of samples (*n*) was lower than the overall number analyzed because clinical data were unavailable for these samples.

Supplementary Table 3: Cox proportional hazard model of clinical parameters and BEND5 methylation levels associated with 5-year overall survival in patients with colorectal cancer from TCGA data set

	Multivariate analysisa		
**Variable**	**HR**	**95% CI**	***P*-value[C]**
Sex	1.216	0.635–2.327	0.556
Age	1.036	1.008–1.065	0.012[*]
Tumor type	2.674	1.145–6.244	0.023[*]
Race	1.103	0.454–2.679	0.829
Stage	2.836	1.864–4.314	< 0.001[***]
BEND5[b] methylation	2.042	1.061–3.933	0.033[*]

[a]These results were analyzed by the Cox regression model.
[b]BEND5 was considered hypermethylated at an average β value of > 0.5.
[c]*$P < 0.05$; **$P < 0.01$; ***$P < 0.001$.

Supplementary Table 4: The predicted BEND5 binding sites in promoters of genes

Gene name	Sequence (5′ to 3′)
BCL2	TTAATCAGAAGA
CCNB1	TCCAATTGCTGA
CCND1	CCAATTAGGAA
CCND2	TCCTTAATAACGAGA
PCNA	CCAATGGGAG
CDKN1A	TATTCAGGAGA

Supplementary Table 5: List of primers sequences and their reaction conditions used in the present study

Real-time RT-PCR[a]		Sequence (5′ to 3′)	Probe	Tm (°C)	Size (bp)
GAPDH	Forward	AGCCACATCGCTCAGACAC	#60	60	66
	Reverse	GCCCAATACGACCAAATCC			
BEND5	Forward	AGCCACATCGCTCAGACAC	#1	60	95
	Reverse	GCCCAATACGACCAAATCC			
BCL2	Forward	ACAGAGGATCATGCTGTACTTAAAAA	#6	60	96
	Reverse	TTATTTCATGAGGCACGTTATTATTAG			
CCNB1	Forward	ACATGGTGCACTTTCCTCCT	#18	60	103
	Reverse	AGGTAATGTTGTAGAGTTGGTGTCC			
CCND1	Forward	GACCTTCGTTGCCCTCTGT	#49	60	105
	Reverse	CTCAGGTTCAGGCCTTGC			
CCND2	Forward	GGACATCCAACCCTACATGC	#49	60	71
	Reverse	CGCACTTCTGTTCCTCACAG			
PCNA	Forward	TGGAGAACTTGGAAATGGAAA	#69	60	95
	Reverse	GAACTGGTTCATTCATCTCTATGG			
CDKN1A	Forward	TCACTGTCTTGTACCCTTGTGC	#32	60	128
	Reverse	GGCGTTTGGAGTGGTAGAAA			
ACTIN	Forward	CCAACCGCGAGAAGATGA	#64	60	97
	Reverse	CCAGAGGCGTACAGGGATAG			
**Quantitative methylation-specific PCR**					
ACTB	Forward	TGGTGATGGAGGAGGTTTAGTAAGT		60	132
	Reverse	AACCAATAAAACCTACTCCTCCCTTAA			
	TagMan probe	ACCACCACCCAACACACAATAACAAACACA			
BEND5	M-Forward	GTTTGGGTTTTGGGGAGTC		63	109
	M-Reverse	GATCGAACAACTCAACCCG			
	TagMan probe	CGAAAATAAAAATCCGACGA			
GAPDH	Forward	GGT AGT TGT TAG GAA AGT TTG TTG G		60	138
	Reverse	TAA TCT AAA AAA AAC ATC ACC CAA A			

[a]RT-PCR: reverse-transcriptase polymerase chain reaction.
